# Acute necrotizing calculous cholecystitis after treatment with ceftriaxone in an elderly patient: a case report

**DOI:** 10.1186/s40792-022-01450-5

**Published:** 2022-05-18

**Authors:** Tsunehiko Shigemori, Ichiro Imoto, Yasuhiro Inoue, Ryo Nishiwaki, Natsuko Sugimasa, Tetsuya Hamaguchi, Midori Noji, Kenji Takeuchi, Yoshiyuki Ito, Taro Yasuma, Esteban C. Gabazza, Toshio Kato

**Affiliations:** 1 Department of Surgery, Doshinkai Tohyama Hospital, Minami-Shinmachi 17-22, Tsu, Mie 514-0043 Japan; 2 Digestive Endoscopy Center, Doshinkai Tohyama Hospital, Minami-Shinmachi 17-22, Tsu, Mie 514-0043 Japan; 3 Department of Immunology, Mie University Faculty and Graduate School of Medicine, Edobashi 2-174, Tsu, Mie 514-8507 Japan

**Keywords:** Ceftriaxone (CTRX), Pseudolithiasis, Acute necrotizing calculous cholecystitis, Cholecystectomy

## Abstract

**Background:**

Ceftriaxone, a third-generation cephalosporin antibiotic with a long plasma half-life, is widely used to treat various infections. The use of ceftriaxone can sometimes induce biliary sludge or stone formation. Although most cases of ceftriaxone-induced pseudolithiasis are asymptomatic or mild and resolve with discontinuation of the drug, we experienced an elderly case of severe acute necrotizing calculous cholecystitis after administration of ceftriaxone.

**Case presentation:**

A 72-year-old male patient was admitted to our hospital because of acute diverticulitis in ascending colon. Ceftriaxone was administered at a dose of 2 g/day for 6 days. Although he recovered after therapy, he was readmitted about 2 weeks later because of severe pain with rebound tenderness in the right upper quadrant. An abdominal imaging study revealed stones and sludge in the gallbladder that were not observed before starting ceftriaxone therapy. Therefore, antibiotic treatment with flomoxef 2 g/day was indicated. However, on the fifth day of readmission, the peritoneal irritation symptoms in the right upper quadrant worsened, and elevated inflammatory response and liver dysfunction were observed. Cholecystectomy was performed based on these findings. The resected inflamed gallbladder showed acute necrotizing cholecystitis with sand granular gallstones. A comparative analysis of the infrared spectroscopic pattern of the composition of gallstones collected during surgery with that of the ceftriaxone powder revealed that both have very similar infrared spectroscopic patterns.

**Conclusions:**

Ceftriaxone-related pseudolithiasis is generally reversible and mainly observed in children. Here, we report a rare case of ceftriaxone-related acute necrotizing cholecystitis in an elderly patient. We confirmed that the stones in the gallbladder are composed of ceftriaxone. The older age, dehydration, fasting, and long-time bed rest during the administration of high-dose ceftriaxone were the potential risk factors for gallstone formation.

## Background

Ceftriaxone (CTRX) is a third-generation cephalosporin with broad-spectrum antibacterial activity against Gram-positive, Gram-negative, aerobic and anaerobic bacteria. CTRX is an antibiotic with a long half-life in plasma that is widely used to treat various infectious diseases. In 1986, Schaad et al. reported the first case of transient formation of precipitations in the gallbladder after ceftriaxone therapy [1]. This clinical observation was named 'pseudolithiasis' because the gallstone-like appearance spontaneously disappears after the cessation of CTRX [2]. CTRX-related pseudolithiasis is frequent in children [3]. However, recent reports have shown a high incidence of CTRX-induced pseudolithiasis in older people [4].

Rare but severe complications that required biliary drainage and surgical resection have been reported. In addition, necrotizing cholecystitis after CTRX therapy has been reported in pediatric patients [5–7] and rarely in adults [8]. Here, we report a very rare case of cholecystectomy for acute necrotizing calculous cholecystitis in an elderly male patient after treatment with CTRX.

## Case presentation

**Fig. 1:**
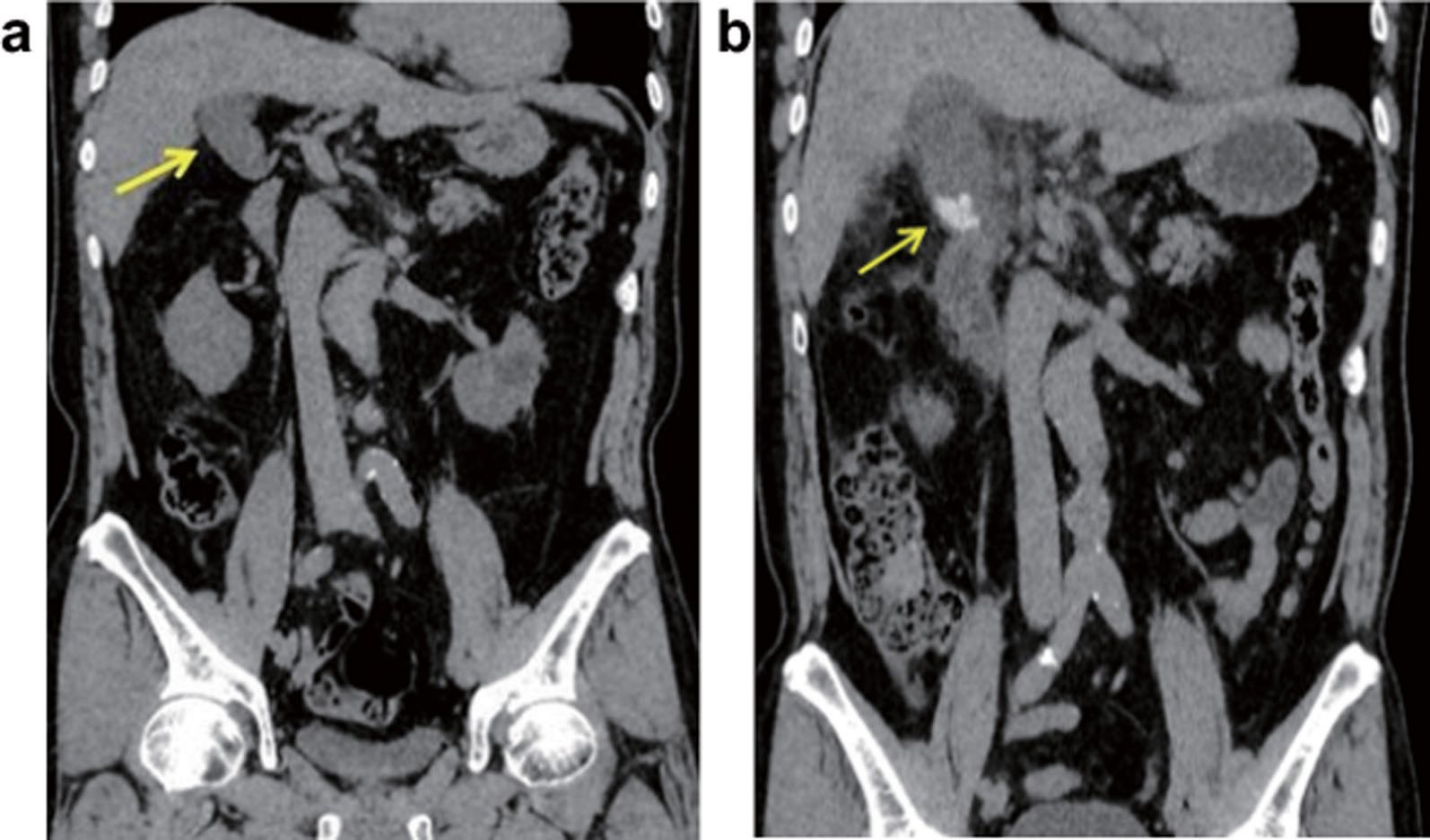
Computed tomography study. Coronal CT section before (**a**) and after (**b**) the administration of ceftriaxone. Gallbladder stone was not detected before ceftriaxone (**a**). However, an inflamed gallbladder (arrow) with stones can be visualized after ceftriaxone administration (**b**)

A 72-year-old man with a history of angina pectoris was admitted with severe pain and tenderness in the right upper quadrant. The body weight was 64 kg, and the body mass index was 23.1. Computed tomography (CT) showed many diverticula and wall thickening in ascending colon and hepatic flexure. He was admitted to our hospital with the diagnosis of diverticulitis. Fasting and CTRX at a dose of 2 g/day for 6 days were indicated. CT revealed no signs of gallstone or cholecystitis before starting CTRX therapy (Fig. 1a). The patient was discharged after a good response to treatment. Twelve days after discharge, the patient had severe pain in the abdominal right upper quadrant with rebound tenderness and was readmitted to our hospital. The blood tests showed an increased inflammatory response characterized by leukocytosis (white blood cell count 11,400/μl), neutrophilia (88.2%), and high level of C-reactive protein (12.1 mg/dl). There were also signs of polycythemia (hemoglobin 18.0 g/dl, hematocrit 55.2%) probably due to dehydration and mild liver dysfunction (alanine aminotransferase 34 IU/L, aspartate aminotransferase 37 IU/L). The biliary enzymes and renal function (blood urea nitrogen/creatinine 16.6/0.84 mg/dl) were normal.

**Fig. 2:**
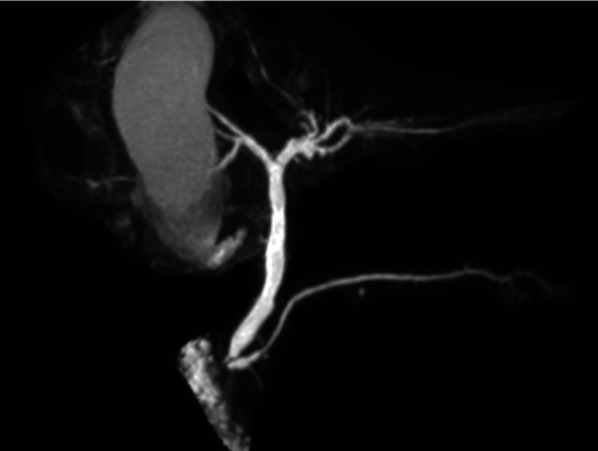
Magnetic resonance cholangiopancreatography study after the administration of ceftriaxone. The base of the swollen gallbladder shows biliary sludge and stones. The common bile duct shows mild dilatation, but with no stones

Abdominal ultrasonography performed before starting CTRX showed no stones in the gallbladder. However, the ultrasonography upon readmission showed enlargement and thickening of the gallbladder wall and numerous biliary sand-like stones in the neck. The abdominal CT showed dilated gallbladder containing calcified gallstones after CTRX therapy (Fig. 1b). A magnetic resonance cholangiopancreatography revealed a mild dilation of the common bile duct, but no obvious common bile duct stones (Fig. 2).

**Fig. 3:**
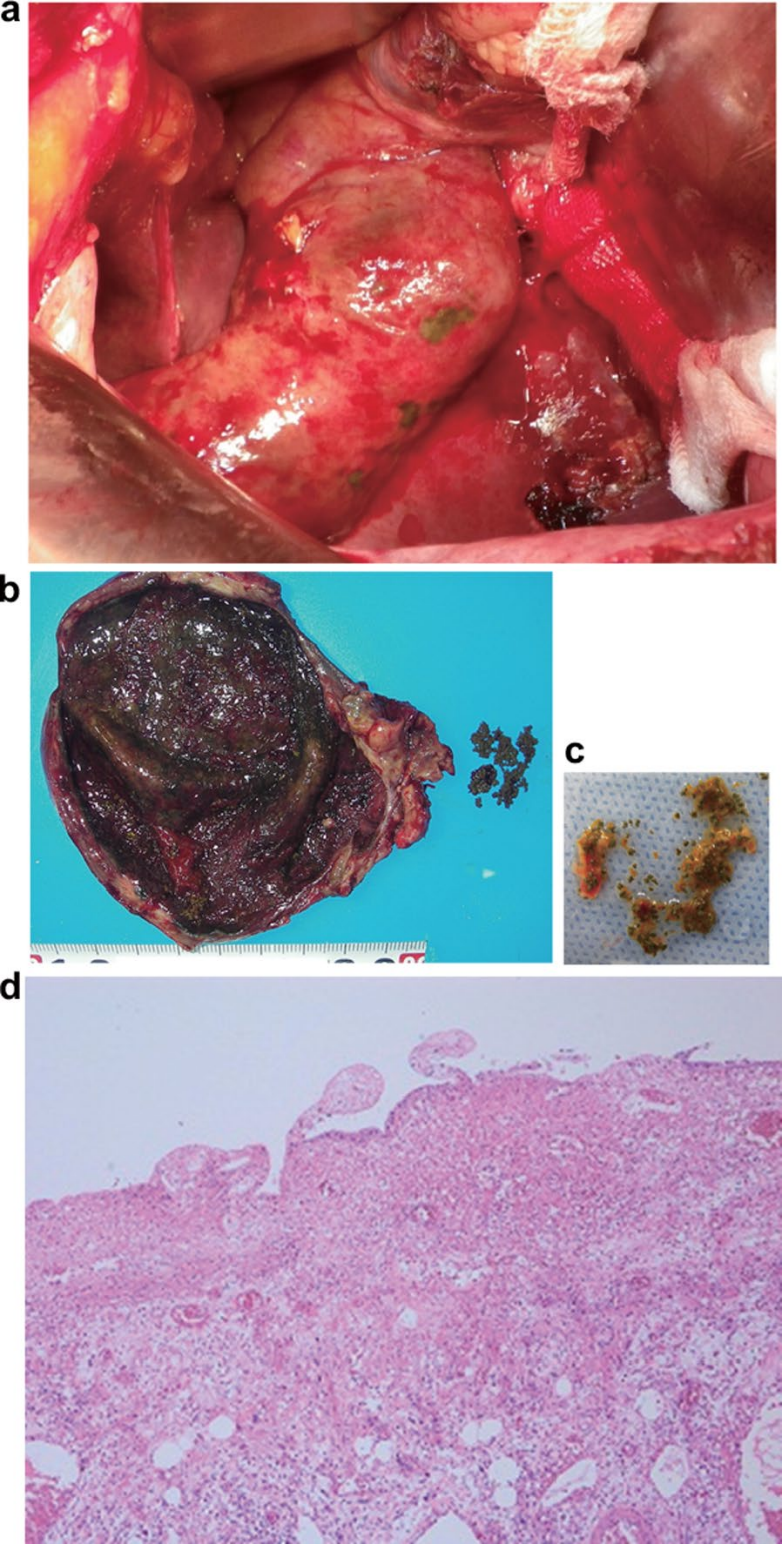
Intraoperative findings. Intra-abdominal view (**a**). Macroscopic findings show a distended gallbladder with a dark-red surface (**b**). Also, the gallbladder shows a thickened wall and necrotic mucosa with many sand granular stones inside (**b**, **c**). Microscopic findings include necrosis, edema, and moderate infiltration of inflammatory cells, including neutrophils and eosinophils (**d**)

**Fig. 4:**
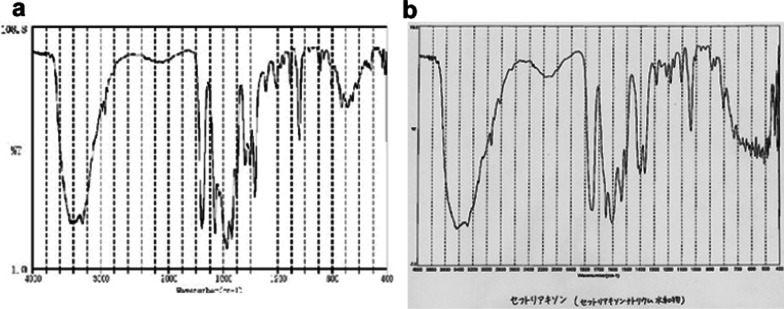
Comparative analysis of gallstones and ceftriaxone powder using infrared spectroscopy. Gallstones (**a**) and ceftriaxone powder (**b**) show very similar patterns of infrared spectroscopy

The diagnosis was acute cholecystitis with gallstones. Intravenous drip infusion, fasting, and flomoxef (FMOX) 2 g/day were indicated. However, on the 5th day of hospitalization, peritoneal irritation symptoms in the right upper quadrant worsened, and the blood test showed an increased inflammatory response (C-reactive protein 22.4 mg/dl) and liver dysfunction (alanine aminotransferase 135 IU/L/aspartate aminotransferase 126 IU/L). The patient underwent cholecystectomy. Intraoperative findings (Fig. 3a) revealed a tense and dark-red gallbladder. The resected gallbladder (Fig. 3b, c) showed wall thickening and the presence of sandy stones. Histopathological findings revealed necrosis, edema, and moderate inflammatory cell infiltration with neutrophils and eosinophils, indicating necrotizing cholecystitis. However, the histopathological study showed no malignant findings (Fig. 3d). No stone was detected in the common bile duct. A comparative analysis of the infrared spectroscopic pattern of the composition of gallstones collected during surgery with that of the CTRX powder revealed that both have very similar infrared spectroscopic patterns (Fig. 4). The patient had a good postoperative course and was discharged on the 19th day of hospitalization.

## Discussion

CTRX is an antibiotic with a long plasma half-life because approximately 85% to 95% of CTRX is bound to albumin after intravenous administration. Renal (55%) and biliary (45%) excretions are the major routes for CTRX elimination from the body [9]. The concentration of excreted CTRX in bile is 20 to 150 times higher than in serum. CTRX binds to calcium ions with high affinity leading to CTRX calcium salt and biliary sludge formation [10]. The precise underlying pathogenic mechanism of this adverse effect is not completely clear. Several mechanisms have been proposed. For example, it has been reported that CTRX may be involved in gallstone formation by decreasing gallbladder contractility in experimental models of guinea pigs [11]. In addition, UDP-glucuronosyltransferase 1A1 gene polymorphism has been reported to predispose gallstone formation in the presence of CTRX [12].

Several other potential risk factors for pseudolithiasis have also been reported. Murata et al. reported that among 60 pediatric patients treated with CTRX, 11 patients (18.3%) developed pseudolithiasis, the main risk factors being fasting and long-term bed rest [13]. These findings suggest that special attention should be paid to the degree of oral intake and patient activity when CTRX is prescribed. Additional predisposing factors of pseudolithiasis in elderly patients include the dose of CTRX, duration of treatment, and the presence of renal dysfunction [4, 14]. In our current case, old age, fasting, dehydration, long-term bed rest, and high-dose CTRX were the predisposing factors of CTRX-related pseudolithiasis. Although pseudolithiasis is generally asymptomatic and reversible, our present case developed acute necrotizing calculous cholecystitis, a rare and severe form of pseudolithiasis in the elderly. The precise mechanism of this severe complication is unclear.

## Conclusion

This report describes a rare case of acute necrotizing cholecystitis associated with CTRX therapy in adults. The lesson we learned from this case is that in addition to mild and reversible cases of CTRX-related pseudolithiasis, there may be patients with pseudolithiasis with severe complications that require surgical treatment. In addition, we confirmed that the precipitates in the gallbladder are related to CTRX.

## Data Availability

The authors declare that all the data are described in this article and available upon reasonable request.

## Abbreviations

CT: Computed tomography
CTRX: Ceftriaxone
UDP-glucuronosyltransferase: Uridine 5’-diphospho-glucuronosyltransferase
